# Inhibitory effect of quercetin on titanium particle induced endoplasmic reticulum stress related apoptosis and *in vivo* osteolysis

**DOI:** 10.1042/BSR20170961

**Published:** 2017-08-14

**Authors:** Laibo Zhang, Zhoubin Tian, Wei Li, Xianquan Wang, Zhentao Man, Shui Sun

**Affiliations:** Department of Bone and Joint, Shandong Provincial Hospital Affiliated to Shandong University, Jinan 250021, P.R. China

**Keywords:** apoptosis, endoplasmic reticulum stress, macrophage, quercetin, titanium

## Abstract

Wear particle induced periprosthetic osteolysis is the main cause of aseptic loosening of orthopedic implants. The aim of the present study is to determine the protective effect of quercetin (QUE) against titanium (Ti) particle induced endoplasmic reticulum stress (ERS) related apoptosis and osteolysis. In the present study, RAW264.7 cells were pretreated with different concentrations (40, 80, and 160 μmol/l) of QUE for 30 min and then treated with Ti particle (5 mg/ml) for 24 h. Cell viability and apoptosis were determined using MTT assay and Annexin V-FITC Apoptosis Detection Kit, respectively. Protein and mRNA expressions of ERS-related genes were examined by Western blot and real-time PCR, respectively. The release of inflammatory cytokines was detected by ELISA. Then, a mouse calvarial osteolysis model was established. Histological sections of calvaria were stained with Hematoxylin-Eosin (H&E) or tartrate-resistant acid phosphatase (TRAP). The results showed that Ti particle reduced cell viability and induced apoptosis in RAW264.7 macrophages. The cytotoxic effects of Ti particle were dramatically inhibited by QUE pretreatment. Interestingly, we found that QUE also significantly reduced Ti particle induced up-regulation of the expression levels of protein kinase RNA-like ER kinase (PERK), inositol-requiring enzyme-1 (IRE1), glucose-regulated protein (GRP78), CCAAT/enhancer-binding protein homologous protein (CHOP), caspase-12, and caspase-3 and enhanced the down-regulation of Bcl-2. In addition, QUE decreased Ti particle-induced inflammatory cytokines release from RAW264.7 cells. Moreover, treatment with QUE markedly decreased osteoclast number. In a mouse calvarial osteolysis model, QUE inhibited Ti particle induced osteolysis *in vivo* by inhibiting osteoclast formation and expressions of ERS-related genes. In conclusion, QUE can protect RAW264.7 cells from Ti particle induced ERS-related apoptosis and suppress calvarial osteolysis *in vivo*.

## Introduction

Total joint replacement (TJR), which is by the implantation of a permanent in-dwelling artificial prosthesis, is a highly successful procedure for promoting the agility in patients with joint dysfunction [1]. It has been proved that titanium (Ti) components have been widely used for joint replacement [2]. However, aseptic loosening due to periprosthetic osteolysis, induced by the adverse biological responses to wear particles, can damage the efficacy and longevity of the prosthetic components [3,4]. The particles generated from the mechanical wear of prosthetic components can be phagocytosed by macrophages and other inflammatory cells [5]. During the pathological process, the macrophages activated by Ti particles release proinflammatory cytokines including interleukin (IL)-6, IL-1β and tumor necrosis factor α (TNF-α). [6,7]. In addition, these cytokines lead to an imbalance in bone metabolism, promote osteoclastogenesis, and stimulate mature osteoclasts to absorb the adjacent bone [8,9]. As no effective drug therapy is currently available to prevent osteolysis, more studies are required.

Recently, endoplasmic reticulum stress (ERS) has attracted particular interest due to its role in inflammatory responses under pathological conditions [10]. Besides, ERS is a novel pathway of cellular apoptosis [11]. Glucose-regulated protein (GRP78) is an endoplasmic reticulum chaperone and is used for monitoring ERS [12]. In the early stages, ERS is alleviated by the activation of unfold protein response (UPR). The following signal transduction pathways are involved in the UPR: protein kinase RNA-like ER kinase (PERK) and inositol-requiring enzyme-1 (IRE1). If the ERS is not alleviated by the UPR, persistent and severe ERS leads to cellular apoptosis. The apoptosis is initiated by up-regulating the ERS-related proapoptotic marker CCAAT/enhancer-binding protein homologous protein (CHOP) [13].

Quercetin (QUE) is one of the major flavonoids, ubiquitously distributed in plants [14]. QUE has been reported to have anti-inflammatory effects, which are mediated through the suppression of proinflammatory cytokines [15]. Moreover, intense exercise induced ERS and inflammation can be attenuated by QUE [16]. However, the protective effect of QUE against Ti particle induced ERS remains unclear.

We designed the current study to determine the potential protective effect of QUE against Ti particle induced ERS-related apoptosis and osteolysis.

## Materials and methods

### Cell culture

The murine macrophage cell line RAW264.7 was purchased from the American Type Culture Collection (Manassas, U.S.A.). The cells were cultivated in Dulbecco’s modified Eagle’s medium (DMEM; Gibco, U.S.A.) supplemented with 10% FBS (Gibco, U.S.A.), 100 U/ml penicillin, and 100 μg/ml streptomycin. Cells were grown in a humidified incubator at 37°C in 5% CO_2_.

### Preparation of Ti particles

Titanium alloy particles (Ti-6Al-4V) (mean particle size: 2.3 μm, size range: 0.1–68 μm) were purchased from the Zimmer Corporation (Warsaw, U.S.A.). Ti particles were prepared as previously described [17]. The particles were sterilized at 180°C for 6 h, followed by washing with 70% ethanol for 48 h to remove endotoxin and confirmed endotoxin-free using a commercial detection kit (Sigma, U.S.A.). Ti particles were sonicated and vortexed before treatment.

### Cell treatment

Cells (1 × 10^5^/ml) were seeded in culture plates. They were divided into five groups: control group (vehicle DMSO); Ti particle (5 mg/ml) group; Ti + high-dose QUE (160 μmol/l) group; Ti + medium-dose QUE (80 μmol/l) group; Ti + low-dose QUE (40 μmol/l) group. In the Ti + QUE groups, cells were subjected to QUE at a final concentration of 40, 80, or 160 μmol/l for 30 min, followed by treatment with Ti particle (5 mg/ml) for an additional 24 h. Subsequently, cells were harvested for further analysis.

### Cell viability

Cell viability was determined by MTT assay. RAW264.7 macrophage cells were seeded at 5 × 10^5^ cells per well and incubated with QUE at various concentrations (40, 80, and 160 μmol/l) for 24 h at 37°C. After incubation, 20 μl of MTT solution (5 mg/ml) was added to each well and the cells were incubated for 4 h. The formazan crystals were dissolved in 200 μl of DMSO. Absorbance was determined at 570 nm. The results were expressed as a percentage of surviving cells over control cells.

### Flow cytometry

To detect apoptotic cells, 1 × 10^5^/ml RAW264.7 macrophage cells were plated in 24-well plates. After the treatment, cells were harvested and analyzed for cell apoptosis by Annexin-V and propidium iodide (PI) staining, using FITC Annexin-V apoptosis detection kit (Life Technology) according to the manufacturer’s instructions. Then, the cells were detected using flow cytometry, and the data were analyzed using FlowJo7.6.1.

### Quantitative real-time PCR

RNA was prepared with TRIzol reagent (Invitrogen, U.S.A.). First-strand cDNA was synthesized in a 20-μl reaction volume using SuperScript III reverse transcriptase (Life Technologies). Q-PCR was performed in triplicate to amplify all targets by using a FastStart DNA Master SYBR Green I Kit (Roche, U.S.A.) according to the manufacturer’s instructions and a Roche Light Cycler Thermocycler. Gene expression data were normalized to *GAPDH* mRNA levels. Primers are listed in Table 1.

**Table 1:** Primer information for qPCR

Gene	Primers	Product size (bp)
PERK	F: 5′-GGGTGGAAACAAAGAAGAC-3′	215
	R: 5′-CAATCAGCAACGGAAACT-3′	
IRE1	F: 5′-GCGATGGACTGGTGGTAACT-3′	184
	R: 5′-GTTTGCTCTTGGCCTCTGTC-3′	
GRP78	F: 5′-GGCGTGAGGTAGAAAAGG-3′	196
	R: 5′-ATGGTAGAGCGGAACAGG-3′	
CHOP	F: 5′-ACCTTCACTACTCTTGACCCT-3′	129
	R: 5′-TCTTCCTCCTCTTCCTCCT-3′	
Caspase-12	F: 5′-CTGGCCCTCATCATCTGCAACAA-3′	173
	R: 5′-CGGCCAGCAAACTTCATTAAT-3′	
Caspase-3	F: 5′-GGAGCTGGACTGTGGCATTGA-3′	94
	R: 5′-CAGTTCTTTCGTGAGCATGGA-3′	
Bcl-2	F: 5′-ACAGAGGGGCTACGAGTG-3′	158
	R: 5′-GGCTGGAAGGAGAAGATG-3′	
TRAP	F: 5′-GCTACTTGCGGTTTCACTATGGA-3′	201
	R: 5′-TGGTCATTTCTTTGGGGCTTATCT-3′	
RANK	F: 5′-AGATGTGGTCTGCAGCTCTTCCAT-3′	124
	R: 5′-ACACACTTCTTGCTGACTGGAGGT-3′	
GAPDH	F: 5′-GAAGGTGAAGGTCGGAGTC-3′	166
	R: 5′-GAAGATGGTGATGGGATTTC-3′	

Abbreviations: RANK, receptor activator of NF-κB; TRAP, tartrate-resistant acid phosphatase.

### ELISA for cytokines

TNF-α, IL-6, and IL-1β levels in culture medium were quantitated using monoclonal anti-TNF-α, IL-6, or IL-1β antibodies according to the manufacturer’s instructions (R&D Systems, U.S.A.).

### Tartrate-resistant acid phosphatase staining

For differentiation of RAW 264.7 cells into osteoclasts, cells were seeded in 96-well plates (10^4^ cells/well). The tartrate-resistant acid phosphatase (TRAP) staining kit (Sigma) was used to evaluate TRAP expression. TRAP^+^ cells with more than three nuclei were counted as osteoclasts using optical microscopy, and ImagePro Plus was used to quantitate the data.

### Mouse calvarial osteolysis model and staining

The experimental design was approved by the Ethics Committee of Shandong Provincial Hospital affiliated to Shandong University. All animals’ experimental procedures were performed according to the guidelines of the Care and Use of Laboratory Animals by the National Institute of Health, China.

The calvarial osteolysis model was established as published previously [18]. In brief, 24 healthy 6-week-old male BALB/C mice (weighing 18 ± 5 g) were equally randomized into four groups with six rats in each group: control group, Ti group, Ti  +  QUE (50 mg/kg per day) group and Ti  +  QUE (100 mg/kg per day) group. In Ti + QUE groups, mice were fed with a daily dose of 50 or 100 mg  of QUE per kg of body weight, respectively. QUE was fed from the third day before the operation until killed.

To inject Ti particles, mice were anesthetized with an intraperitoneal injection of pentobarbital. The surgical area was manually depilated and disinfected. A 0.5 -cm sagittal incision was made and the periosteum remained intact. Subsequently, a 25-gauge needle was used to inject 100 μl of 30 mg Ti particles resuspended in PBS directly over the calvarial bone and periosteum. Ten days after the operation, mice were killed and the calvaria were excised, fixed, and decalcified in EDTA. Histological sections of calvaria were stained with Hematoxylin-Eosin (H&E) or TRAP according to the manufacturers’ instructions.

### Western blot

Proteins were extracted in 100  µl of SDS lysis buffer (10 mM EDTA, 25  mM Tris/HCl, pH 7.4, 95  mM NaCl, 2% SDS) with protease inhibitors (Sigma). Protein content was measured by the BCA method (Pierce) according to the manufacturer’s instructions. Total proteins (40  µg) were separated by SDS/PAGE (10% gel). Then the protein was blotted on to PVDF membrane (Bio–Rad) at 70 V for 65 min. Subsequently, the membranes were incubated with primary antibodies, including anti-PERK antibody (Cell Signaling), anti-IRE1 antibody (R&D Systems), anti-GRP78 antibody (Santa Cruz Biotechnology), anti-CHOP antibody (R&D Systems), anti-cleaved caspase-12 antibody (R&D Systems), anti-cleaved caspase-3 antibody (Cell Signaling), anti-Bcl-2 antibody (R&D Systems), and anti-GAPDH antibody (Sigma–Aldrich) overnight at 4 °C. Then the membranes were incubated with secondary antibody conjugated to horseradish peroxidase (Boster, Wuhan, China) for 1  h at room temperature. The chemiluminescent signal was detected by the ECL Detection Reagents (Amersham).

### Statistics analysis

The data are expressed as the mean ± S.D. Statistical analysis was performed with SPSS 16.0. Comparisons between the two groups were evaluated by Student’s *t* test. A *P*-value<0.05 was considered statistically significant.

## Results

### QUE inhibits Ti particle induced cell death

Cells were incubated with various concentrations of QUE (0, 40, 80, 160 μmol/l) for 24 h. As shown in Supplementary Figure S1A, no reduction in cell viability was observed when treated with QUE, demonstrating no detectable cytotoxicity of the drug. However, pretreated with various concentrations of QUE (40, 80, 160 μmol/l) alleviated Ti particle induced reduction in cell viability (Supplementary Figure S1B).

### QUE decreases Ti particle induced ERS-related apoptosis of RAW264.7 cells

**Figure 1:**
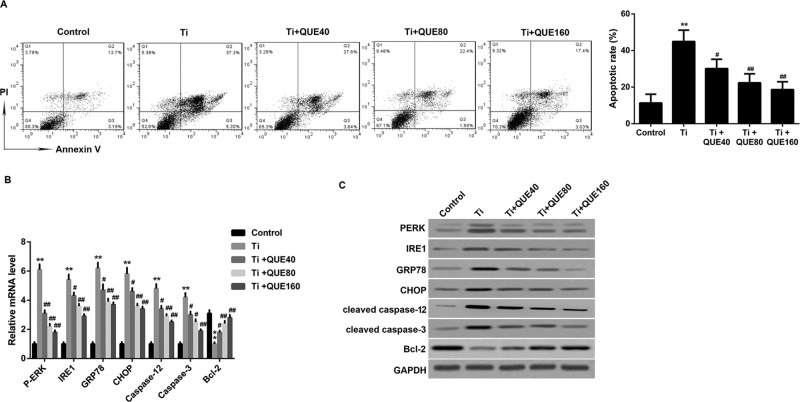
QUE decreases Ti particle induced ERS-related apoptosis in RAW264.7 cells (**A**) The ratio of apoptotic cells stained with Annexin V-FITC and PI was detected by flow cytometry analysis (*n*=3). (**B**) qRT-PCR analysis of *PERK, IRE1, GRP78, CHOP, caspase-12, caspase-3, and Bcl-2* mRNA in the RAW264.7 cells under different treatments (*n*=3). (**C**) PERK, IRE1, GRP78, CHOP, cleaved caspase-3, cleaved caspase-12, and Bcl-2 protein levels in RAW 264.7 macrophages were determined by Western blot (*n*=3). **P<0.01 compared with control group; ^#^*P*<0.05 and ^##^*P*<0.01 compared with Ti group. Abbreviations: qRT-PCR, quantitative real-time PCR; QUE40, 40 μmol/l QUE; QUE80, 80 μmol/l QUE; QUE160, 160 μmol/l QUE.

To evaluate the effect of QUE on cell apoptosis, Annexin V/PI staining was used. As shown in Figure 1A, Ti particle induced cell apoptosis of RAW264.7 cells. However, QUE had a significant anti-apoptotic effect on Ti particle induced cell apoptosis.

To determine whether anti-apoptosis induced by QUE was related to ERS signaling pathways, the changes in apoptosis-related and ERS-related genes were tested by Q-PCR and Western blot. Compared with the control group, the increased PERK, IRE1, GRP78, CHOP, caspase-12, and caspase-3 levels, while decreased Bcl-2 levels were observed in the Ti group (Figure 1B,C). However, QUE dose-dependently decreased PERK, IRE1, GRP78, CHOP, caspase-12, and caspase-3 levels, and increased the Bcl-2 levels in the Ti particle treated RAW264.7 cells (Figure 1B,C).

### QUE decreases Ti particle induced inflammatory cytokines release from RAW264.7 cells

**Figure 2:**
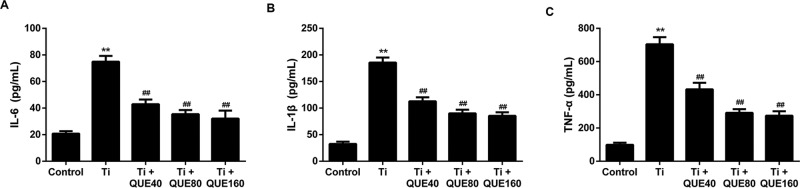
QUE decreases Ti particle induced inflammatory cytokines release from RAW264.7 cells (**A**) IL-6, (**B**) IL-1β, and (**C**) TNF-α levels in cell culture supernatants from RAW264.7 cells under different treatments (*n*=3). **P<0.01 compared with control group; ^##^*P*<0.01 compared with Ti group. Abbreviations: QUE40, 40 μmol/l QUE; QUE80, 80 μmol/l QUE; QUE160, 160 μmol/l QUE.

To evaluate the effect of QUE on Ti particle induced inflammatory cytokines release, RAW264.7 cells were treated with QUE (40, 80, 160 μmol/l). QUE dramatically inhibited Ti particle induced IL-6, IL-1β, and TNF-α release from RAW264.7 cells (Figure 2A–C). The results demonstrate that QUE has a dose-dependent inhibitory effect on Ti particle induced inflammatory cytokines release in macrophages.

### QUE inhibits Ti particle induced differentiation of osteoclasts

**Figure 3:**
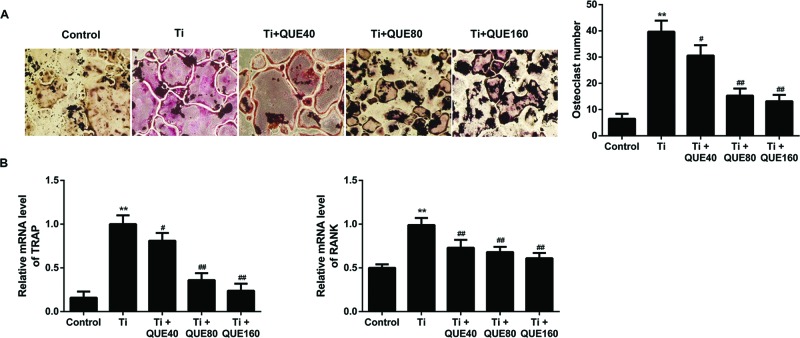
QUE inhibits Ti particle induced differentiation of osteoclasts (**A**) Representative TRAP staining images (left) and quantitative analysis of the number of TRAP^+^ osteoclasts (right), magnification ×100 (*n*=3). (**B**) qRT-PCR analysis of *TRAP* and *RANK* mRNA expression levels in the RAW264.7 cells under different treatments (*n*=3). **P<0.01 compared with control group; ^#^*P*<0.05 and ^##^*P*<0.01 compared with Ti group. Abbreviations: qRT-PCR, quantitative real-time PCR; QUE40, 40 μmol/l QUE; QUE80, 80 μmol/l QUE; QUE160, 160 μmol/l QUE.

To determine whether QUE inhibits osteoclast differentiation, TRAP-positive cells were analyzed by TRAP staining. As shown in Figure 3A, a significant increase in osteoclasts was observed in the RAW264.7 cells treated with Ti particle. Treatment with QUE markedly decreased osteoclast number in a dose-dependent manner. In addition, we analyzed TRAP and receptor activator of NF-κB (RANK) expression in RAW264.7 cells. RANK is present on osteoclast precursors and induces the development and activation of osteoclasts [19]. The mRNA expression levels of TRAP and RANK were up-regulated in Ti group compared with that in control group (Figure 3B). Consistent with its inhibition of osteoclast differentiation, QUE inhibited the mRNA expression levels of TRAP and RANK. These results are the evidence that QUE reduces osteoclast differentiation.

### Inhibitory effect of QUE on Ti particle induced osteolysis in a mouse calvaria model *in vivo*

**Figure 4:**
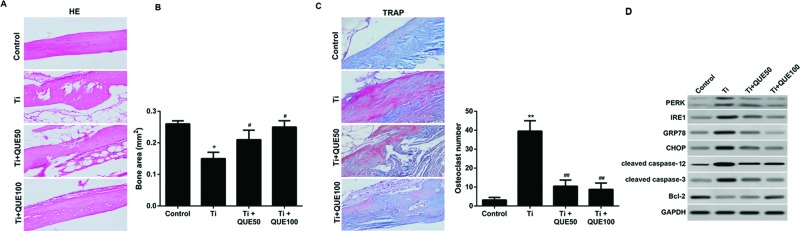
Inhibitory effect of QUE on Ti particle induced osteolysis and ERS-related apoptosis in a mouse calvaria model *in vivo* (**A**) Representative photographs of calvarial histology stained with H&E (left), magnification: ×100 (*n*=3). (**B**) Bone area was measured using a digitalized image analyzer (IMT i-Solution; Korea) (*n*=3). (**C**) Representative TRAP staining images (left) and quantitative analysis of the number of TRAP^+^ osteoclasts (right), magnification: ×40 (*n*=3). (**D**) PERK, IRE1, GRP78, CHOP, cleaved caspase-3, cleaved caspase-12, and Bcl-2 protein levels in the calvaria of mice were determined by Western blot (*n*=3). *P<0.05 and **P<0.01 compared with control group; ^#^*P*<0.05 and ^##^*P*<0.01 compared with Ti group. Abbreviations: QUE50: 50 mg/kg per day QUE; QUE100, 100 mg/kg per day QUE.

Next, we analyzed whether QUE suppressed Ti particle induced osteolysis *in vivo*. Injection of Ti particles into the mouse calvaria notably induced osteolysis compared with control group, while administration of QUE at 50 mg/kg per day or 100 mg/kg per day reduced osteolysis (Figure 4A). Histomorphometric analysis indicated that the average bone area of Ti particle implanted mice was significantly less than that of control group (Figure 4B). In contrast, the bone area of Ti-treated mice was remarkably increased by treatment with QUE (Figure 4B).

To examine whether QUE suppressed Ti particles induced osteoclast formation in the calvaria, TRAP staining was performed. Compared with control group, a significant increase in osteoclasts was observed in the Ti group (Figure 4C). Treatment with QUE at 50 mg/kg per day and 100 mg/kg per day dramatically decreased osteoclasts (Figure 4C).

### QUE inhibits Ti particle induced ERS-related apoptosis *in vivo*

To examine whether ERS-related apoptosis is also decreased by QUE *in vivo*, Western blot was performed. A significant increase in the PERK, IRE1, GRP78, CHOP, cleaved caspase-12, and cleaved caspase-3 protein levels and a significant decrease in Bcl-2 were found in Ti-implanted calvarium compared with the control group (Figure 4D). QUE significantly reduced the protein expression levels of PERK, IRE1, GRP78, CHOP, cleaved caspase-12, and cleaved caspase-3 and enhanced the levels of Bcl-2 in a dose-dependent manner (Figure 4D). These data demonstrate that QUE treatment reduces Ti particle induced ERS-related apoptosis *in vivo*.

## Discussion

Apoptosis may be an important element in understanding the mechanisms of aseptic loosening and periprosthetic osteolysis [20]. Stea et al. [21] demonstrated that the apoptotic cells in the interface membrane, which were collected from revision surgery for aseptic loosening of hip joint prostheses, were mainly macrophages, and apoptosis was correlated with metal wear. Landgraeber et al. [22] found that in particle-induced osteolysis, apoptosis is pathologically increased. Yang et al. [23] proved that apoptosis in macrophages during particle engulfment is partially responsible for osteolysis in aseptic loosening of joint implants. Our results showed that Ti particle reduced cell viability and induced apoptosis in RAW264.7 macrophages, suggesting that apoptosis may be a crucial factor responsible for wear particle induced osteolysis.

Recently, ERS pathway is believed to be a novel apoptotic pathway which is different from mitochondrial pathway and receptor pathway [24]. Increasing evidence suggesting ERS pathway induced apoptosis is related to some pathological processes, such as Alzheimer’s disease [25], diabetes [26], pancreatic cancer [27], and osteoarthritis [28]. However, few studies focussed on whether ERS pathway induced apoptosis was involved in the aseptic loosening. In present study, ERS pathway related markers like PERK, IRE1, GRP78, and CHOP were examined using Western blot and Q-PCR. In mammals, ERS responses can be triggered by activation of three distinct signaling molecules, namely PERK, IRE1, and activating transcription factor 6 (ATF6) [29]. When ERS occurs, PERK and GRP78 dissociate, and activated PERK promotes the phosphorylation of α subunit of translation initiation factor-2 (eIF2α). Phosphorylation of eIF2α decreases protein synthesis, with a few exceptions, such as the activating transcription factor 4 (ATF4) [30]. CHOP is a major target for ATF4, which induces apoptosis in cells [31]. Previous studies have demonstrated that the expression levels of cleaved caspase-3 and GRP78 in macrophages in interface membrane of aseptic loosening were significantly higher than those in the control samples [28]. Our results found that the expressions of PERK, IRE1, GRP78, CHOP, and cleaved caspase-3 were induced, while Bcl-2 was reduced in macrophages in Ti group, indicating that the ERS pathway participated in the process of apoptosis.

The reduction in apoptotic reaction by administration of anti-apoptotic drugs may lead to the prevention of osteolysis. Landgraeber et al. [32] showed that adiponectin decreases wear particle induced osteolysis through its influence on apoptotic and inflammatory pathways. Park et al. [33] found that QUE may suppress the ERS-CHOP pathway induced apoptosis in dopaminergic neurones. Our results demonstrated that QUE inhibited Ti particle induced ERS-related apoptosis and reduced the release of inflammatory cytokines.

The results of the present study also demonstrated that QUE treatment significantly reduced Ti particle induced osteoclast differentiation in a mouse calvaria model. Increasing osteoclastogenesis and the inhibition of bone formation is the primary cause of aseptic loosening and osteolysis [34]. Growing evidence suggests that Ti particle impairs the function of mature osteoblasts as well as inducing osteoclast differentiation [35–37], which is consistent with the current study. In addition, our results strongly indicate that QUE inhibits Ti-induced osteolysis *in vivo* as evident by decreased osteoclast numbers and increased bone area.

In conclusion, QUE inhibits Ti particle induced ERS-related apoptosis and suppresses osteolysis *in vivo*, and therefore may be a therapeutic drug for the prevention and treatment of osteolysis and loosening after TJA.

## Supporting information

**Supplementary Figure:** 

## Abbreviations

ATF4: activating transcription factor 4
Bcl-2: B-cell lymphoma-2
CHOP: CCAAT/enhancer-binding protein homologous protein
eIF2α: α subunit of translation initiation factor-2
ERS: endoplasmic reticulum stress
GRP78: glucose-regulated protein
GAPDH: glyceraldehyde-3-phosphate dehydrogenase
H&E: Hematoxylin-Eosin
IL: interleukin
IRE1: inositol-requiring enzyme-1
NF-κB: nuclear factor-kappa B
PERK: protein kinase RNA-like ER kinase
PI: propidium iodide
QUE: quercetin
Q-PCR: Quantitative real time polymerase chain reaction
RANK: receptor activator of NF-κB
TNF-α: tumor necrosis factor α
TRAP: tartrate-resistant acid phosphatase
UPR: unfold protein response
