# Supplementary figures and images for: Inhibitory effect of quercetin on titanium particle induced endoplasmic reticulum stress related apoptosis and in vivo osteolysis
Source: Biosci Rep. 2017 Aug 14;37(4):BSR20170961. doi: 10.1042/BSR20170961 (PMC5634327; doi:10.1042/BSR20170961)

**A**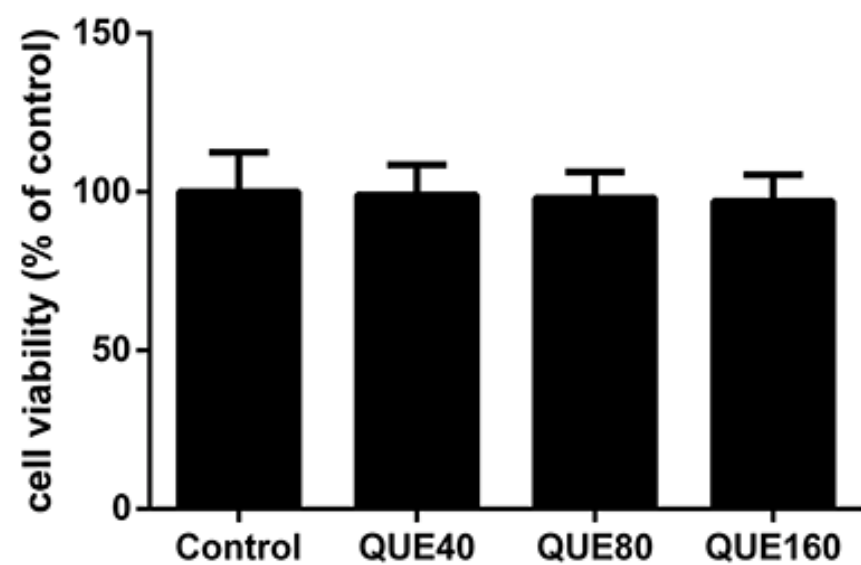**B**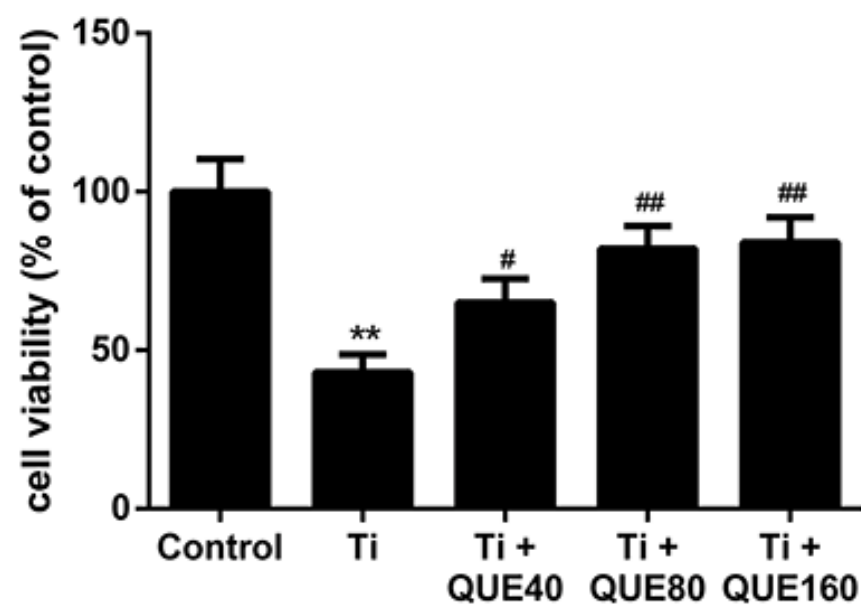

Supplement: Supplementary file 1 [file bsr-37-bsr20170961-s1.pdf]
